# The Flavonoid Quercetin Ameliorates Liver Inflammation and Fibrosis by Regulating Hepatic Macrophages Activation and Polarization in Mice

**DOI:** 10.3389/fphar.2018.00072

**Published:** 2018-02-09

**Authors:** Xi Li, Qianwen Jin, Qunyan Yao, Beili Xu, Lixin Li, Shuncai Zhang, Chuantao Tu

**Affiliations:** ^1^Department of Geriatrics, Zhongshan Hospital, Fudan University, Shanghai, China; ^2^Department of Gastroenterology and Hepatology, Zhongshan Hospital, Fudan University, Shanghai, China; ^3^Shanghai Institute of Liver Diseases, Shanghai, China

**Keywords:** hepatic fibrosis, quercetin, macrophages, polarization, Notch1, hepatic stellate cells (HSCs)

## Abstract

At present, there are no effective antifibrotic drugs for patients with chronic liver disease; hence, the development of antifibrotic therapies is urgently needed. Here, we performed an experimental and translational study to investigate the potential and underlying mechanism of quercetin treatment in liver fibrosis, mainly focusing on the impact of quercetin on macrophages activation and polarization. BALB/c mice were induced liver fibrosis by carbon tetrachloride (CCl_4_) for 8 weeks and concomitantly treated with quercetin (50 mg/kg) or vehicle by daily gavage. Liver inflammation, fibrosis, and hepatic stellate cells (HSCs) activation were examined. Moreover, massive macrophages accumulation, M1 macrophages and their related markers, such as tumor necrosis factor (TNF)-α, interleukin (IL)-1β, IL-6, and monocyte chemotactic protein-1 (MCP-1) in livers were analyzed. *In vitro*, we used Raw 264.7 cells to examine the effect of quercetin on M1-polarized macrophages activation. Our results showed that quercetin dramatically ameliorated liver inflammation, fibrosis, and inhibited HSCs activation. These results were attributed to the reductive recruitment of macrophages (F4/80^+^ and CD68^+^) into the liver in quercetin-treated fibrotic mice confirmed by immunostaining and expression levels of marker molecules. Importantly, quercetin strongly inhibited M1 polarization and M1-related inflammatory cytokines in fibrotic livers when compared with vehicle-treated mice. *In vitro*, studies further revealed that quercetin efficiently inhibited macrophages activation and M1 polarization, as well as decreased the mRNA expression of M1 macrophage markers such as *TNF*-α, *IL*-1β, *IL-6*, and nitric oxide synthase 2. Mechanistically, the inhibition of M1 macrophages by quercetin was associated with the decreased levels of Notch1 expression on macrophages both *in vivo* and *in vitro*. Taken together, our data indicated that quercetin attenuated CCl_4_-induced liver inflammation and fibrosis in mice through inhibiting macrophages infiltration and modulating M1 macrophages polarization via targeting Notch1 pathway. Hence, quercetin holds promise as potential therapeutic agent for human fibrotic liver disease.

## Introduction

Liver fibrosis is a typical wound-healing process triggered by liver injury and inflammation resulting from a wide variety of etiologies, such as chronic virus infection (mainly hepatitis B and C viruses), alcoholic and nonalcoholic steatohepatitis (NASH), drugs, cholestasis, and autoimmune hepatitis (Friedman, 2008; Pellicoro et al., 2014; Tsuchida and Friedman, 2017). Nowadays, hepatic fibrosis is viewed as a dynamic process characterized by the massive excess deposition of extracellular matrix (ECM) in the liver (Friedman, 2008; Tsuchida and Friedman, 2017). It has been generally accepted that resident hepatic stellate cells (HSCs), which become activated and transdifferentiate into myofibroblast-like cells in response to chronic liver injury, are the major source of ECM during the process of liver fibrogenesis (Pellicoro et al., 2014; Seki and Schwabe, 2015; Tsuchida and Friedman, 2017). It has become evident that HSCs activation results from the inflammatory activity of liver immune cells, predominantly macrophages (Pellicoro et al., 2014; Seki and Schwabe, 2015; Li et al., 2017b). Of note, hepatic macrophages can directly mediate the behavior of HSCs and other myofibroblasts by producing a range of cytokines, chemokines, and other soluble mediates (Pellicoro et al., 2014). Additionally, activated myofibroblasts can amplify inflammatory responses by inducing the infiltration of macrophages and further secreting cytokines (Duffield et al., 2005; Pellicoro et al., 2014). Given the critical regulatory role of macrophages in HSCs activation and liver fibrosis, we believe that it provides therapeutic targets promising application in the future.

The prevailing concept indicates that hepatic macrophages can arise either from proliferating resident macrophages, or from circulating bone marrow (BM)-derived monocytes, which are recruited to the injured liver (Duffield et al., 2005; Pellicoro et al., 2014; Wynn and Vannella, 2016). Macrophages are highly plastic cells that can be altered depending on the tissue microenvironment (Tacke and Zimmermann, 2014; Wynn and Vannella, 2016). Its polarization statue to M1 or M2 is often used to characterize macrophages; in which M1 macrophages exhibit an inflammatory phenotype while M2 macrophages are alternatively activated, including an anti-inflammatory phenotype (Beljaars et al., 2014; Sica et al., 2014; Tacke and Zimmermann, 2014; Tosello-Trampont et al., 2016). Moreover, increasing evidence suggests that M1 macrophages activation plays a critical role in liver inflammation and fibrosis (Beljaars et al., 2014; Sica et al., 2014; Tacke and Zimmermann, 2014). Additionally, inflammatory cytokines, including transforming growth factor-β1 (TGF-β1), tumor necrosis factor (TNF)-α, interleukin (IL)-1β, and IL-6, released from these cells trigger local inflammatory responses and perpetuate inflammation as well as HSCs activation (Sica et al., 2014; Tacke and Zimmermann, 2014; Tosello-Trampont et al., 2016). By contrast, emerging evidence suggested that alternative M2 macrophages attenuated hepatic steatosis and inflammation, and have a pivotal role in the resolution of fibrosis (Beljaars et al., 2014; Sica et al., 2014; Tosello-Trampont et al., 2016; Labonte et al., 2017).

Furthermore, macrophage polarization is regulated by several key molecular mechanisms, including epigenetic regulators, transcription factors, posttranscriptional regulators, and some signaling pathways (Sica and Mantovani, 2012; Sica et al., 2014; Wijesundera et al., 2014; Xu et al., 2015a). The switch in phenotypes determines their role in liver inflammation and fibrosis, thus controlling M1/M2 macrophage polarization provides potential targets for antifibrotic therapies (Sica et al., 2014; Xu et al., 2015a; Tacke, 2017). Moreover, it has been reported that the M1 macrophage phenotype was controlled by several molecular signaling or transcription factors, including Notch1 signaling, transducer and activator of transcription 1 (STAT1), and interferon-regulatory factor (IRF) 5 (Lawrence and Natoli, 2011; Sica et al., 2014; Xu et al., 2015a; Tacke, 2017), while IRF4 and STAT6 were shown to specifically regulate M2 macrophage polarization (Lawrence and Natoli, 2011). Thus, potential therapeutic approaches might aim to balance M1/M2 macrophages or to regulate macrophage polarization by targeting key macrophage transcription factors (Lawrence and Natoli, 2011; Sica and Mantovani, 2012; Wan et al., 2014; Labonte et al., 2017).

**FIGURE 1:**
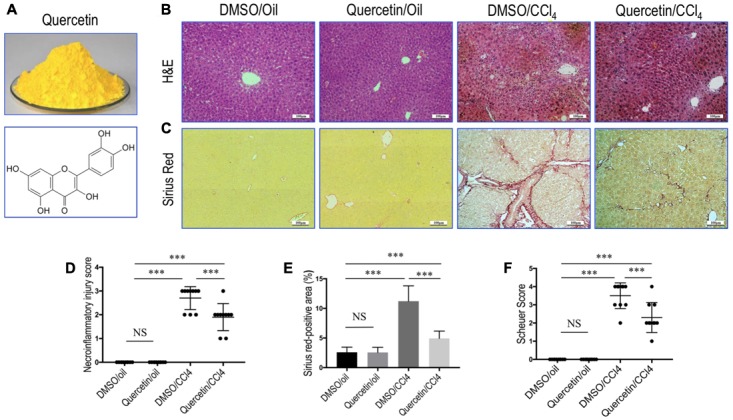
Quercetin inhibited liver inflammation and fibrosis in CCl_4_-treated mice. **(A)** Quercetin powder and chemical structure of quercetin (3,3′,4′,5,7-pentahydroxyflavone). **(B)** Histological examination of liver sections from each group (H&E staining, original magnification: ×100). Scale bar = 100 μm. **(C)** Sirius red staining of liver sections (original magnification: ×100). Scale bar = 100 μm. **(D)** The hepatocellular damage observed in H&E-stained liver sections were analyzed and scored as described under the section “Materials and Methods” (*n* = 10/group). **(E)** Hepatic fibrotic area based on Sirius red staining. **(F)** Assessment of liver fibrosis based on Scheuer’s scoring system. ^∗∗∗^*P* < 0.001; “NS” indicates not significant.

Quercetin (3,3′, 4′,5,7-pentahydroxyflavone; **Figure 1A**) is a well-known flavonoid widely found in many plants and fruits including apples, red grapes, citrus fruit, tomato, onions, and other leafy green vegetables, and a number of berries (Russo et al., 2012; Kim and Park, 2016). Quercetin is known to possess various biological and pharmacological activities including antioxidant, antiviral, anti-inflammatory, anti-proliferative, and antifibrotic effects (Marcolin et al., 2012; Russo et al., 2012; Li et al., 2016a). Indeed, the beneficial effects of quercetin on liver injury and fibrosis have been confirmed by several animal models (Hernandez-Ortega et al., 2012; Marcolin et al., 2012; Li et al., 2016b). Recently, we reported that quercetin inhibited liver inflammation and fibrosis in mice by modulating high mobile group box 1 (HMGB1) and toll-like receptor (TLR) 2/TLR4 signaling pathways (Li et al., 2016b).

However, the precise mechanisms of quercetin on liver fibrosis are incompletely understood. Thus, further studies are needed to define the mechanisms underlying anti-inflammatory and antifibrotic activity of quercetin that hold promise for translation into human treatments. Notably, it has been reported that quercetin attenuated inflammation in human and mouse macrophages *in vitro* upon injury (Overman et al., 2011; Kim and Park, 2016) and reduced mice adipose tissue macrophage infiltration and inflammation in diet-induced obesity (Dong et al., 2014). In the light of these findings, we thus hypothesized that the antifibrotic effects of quercetin should be involved in regulating activation and polarization of hepatic macrophages.

## Materials and Methods

### Reagents and Antibodies

Carbon tetrachloride (CCl_4_), quercetin, dimethyl sulfoxide (DMSO), olive oil, 1,4-diazabicyclo[2.2.2]octane (DABCO), and lipopolysaccharide (LPS; from *Escherichia coli 0727: B8*) were purchased from Sigma Chemical, Co., Ltd. (St. Louis, MO, United States). For *in vivo* and *in vitro* experiments, quercetin was diluted immediately in DMSO solution before administration.

Antibodies used in this study comprised: mouse anti-desmin monoclonal antibody (DakoCytomation, Glostrup, Denmark); rabbit anti-collagen III polyclonal antibody, rabbit anti-collagen IV polyclonal antibody, rabbit anti-CD68 monoclonal antibody, rat anti-F4/80 monoclonal antibody, mouse anti-CD11c polyclonal antibody, mouse anti-IRF5 monoclonal antibody, rabbit anti-Ym-1 monoclonal antibody, rabbit anti-CD163 monoclonal antibody, and rabbit anti-GAPDH monoclonal antibody (Abcam, Cambridge, MA, United States); rabbit anti-IL12a monoclonal antibody, rabbit anti-Notch1 monoclonal antibody, and rabbit anti-β-actin monoclonal antibody (Cell Signaling Technology, Boston, MA, United States).

### Animal Experimental Protocols

Male BALB/c mice (weight 20–22 g) were purchased from the Shanghai Laboratory Animal Research Center (Shanghai, China). All animals were housed in standard cages (23 ± 2°C at a humidity of 55 ± 10%) with a 12 h light/12 h dark cycle. Mice had unrestricted access to food and water. Fibrosis in mice was injected intraperitoneally (i.p.) biweekly for 8 weeks with 0.5 μL/g body weight of CCl_4_, which was dissolved in olive oil at a concentration of 25% v/v (Li et al., 2016b). Fifty-five mice were randomly divided into four groups as described previously [20]. Briefly, Group I (*n* = 10) and Group II (*n* = 15) were given twice weekly injections of olive oil, and received equal volume of DMSO and quercetin (50 mg/kg) by orally, respectively; Group III (*n* = 15) and Group IV (*n* = 15) were injected with CCl_4_ and received quercetin and DMSO, respectively. After 8 weeks of treatment with CCl_4_, mice were sacrificed with pentobarbital, mouse livers were removed to examine for fibrosis. The dose of quercetin for this experiment was based on the previous studies in mice (Hernandez-Ortega et al., 2012; Li et al., 2016a). All animal experiments were performed according to institutional guidelines and regulations and approved by the Animal Care Committee of Fudan University (Shanghai, China).

### Cells Culture and Treatment

Raw 264.7 cells were purchased from Sigma Chemical, Co., Ltd. (St. Louis, MO, United States) and cultured in undifferentiated Raw macrophages conditioned medium. Briefly, Raw 264.7 cells were cultured in T25 flasks in Dulbecco’s Modified Eagle Medium (DMEM) supplemented with 10% fetal bovine serum (FBS), L-glutamine (2 mM), penicillin (50 U/mL), and streptomycin (50 μg/mL) at 37°C and 5% CO_2_. All incubations were performed in cells under the three or four passages (Li et al., 2017b).

In experiments assessing the effects of quercetin on macrophages activation and polarization macrophages, Raw 264.7 cells were polarized by culturing 300,000 cells/well overnight in 24-well plates before replacing the medium with complete culture medium supplemented with M1-differentiated macrophages conditioned medium as described previously (Wan et al., 2014; Tosello-Trampont et al., 2016; Labonte et al., 2017). Briefly, using LPS (100 ng/mL) to induce M1 differentiation. For selective experiments, cells were co-cultured with quercetin (50 μM); and parallel cultures were treated with an equivalent volume of DMSO (0.05%) served as negative controls. Quercetin concentration (50 μM) for macrophage treatment was used in our cell experiments based on previous *in vitro* bioactivity work (Kobuchi et al., 1999; Kim and Park, 2016; Li et al., 2016b). After 24 h of co-culture at 37°C, cells were then washed and harvested by centrifugation for immunofluorescence analysis, RNA harvesting, and protein isolation (Li et al., 2017b). All measurements were performed in triplicate using different batches of wells. Staining and quantitative RT-PCR analysis were performed on three independent experiments.

### Cell Viability Assay

Cell Counting Kit-8 (CCK-8) Assay Kit was used to assess cell viability according to the manufacturer’s instructions as described previously (Li et al., 2017a). Briefly, Raw 264.7 cells were seeded into a 24-well culture plate at a density of 1 × 10^5^ cells/well and incubated with quercetin (50 μM) or vehicle (0.05% DMSO) at 37°C for 0, 12, and 24 h; then, the cells were incubated in 10% CCK-8 that was diluted in normal culture medium at 37°C until the visual color conversion occurred. The absorbance at 450 nm was measured with Flexstation 3 Multimode Microplate Reader (Molecular Device). Experiments were conducted in triplicate independently, and data are presented as means ± *SD*.

### Histopathologic Analysis

The left lobe of liver was excised immediately after mice were euthanized, fixed in 10% neutral buffered formalin, and embedded in paraffin. Four-micron sections were stained with hematoxylin and eosin (H&E) and Sirius red according to standard protocols. The H&E-stained liver tissues were evaluated by an experienced pathologist completely blinded to the identity of the samples, according to criteria in four categories for necroinflammation as previously described (Horrillo et al., 2007), and scored for grade 0 (absent), grade 1 (spotty necrosis), grade 2 (confluent necrosis), and grade 3 (bridging necrosis). Liver fibrosis was assessed by measurement of the Sirius-red positive area, which was measured in six low power (×100) fields per slide using ImageJ 1.49 software (NIH, Bethesda, MD, United States). Fibrosis staging was also classified by a pathologist according to the Scheuer histological scoring system on a scale from 0 to 4 (0 = normal, 4 = cirrhosis) (Scheuer, 1991). The assessment of the preceding scores was uniformly performed under 100× magnification in 10 fields per sample.

### Immunohistochemistry and Quantitative Analysis of Histological Markers

For immunohistochemical analysis, sections of formalin-fixed, paraffin-embedded liver tissue were cut 4 μm, dewaxed, hydrated, and subjected to heat-induced antigen retrieval according to standard protocols as previously reported (Li et al., 2016b, 2017a). Subsequently, sections were treated with 3% hydrogen peroxide for 10 min, then blocked and incubated overnight at 4°C with primary antibodies as follows: rabbit anti-Desmin (1:100), rabbit anti-collagen III (1:100), rabbit anti-collagen IV (1:100), rat anti-F4/80 (1:50), rabbit anti-CD68 (1:100), mouse anti-CD11c (1:100), rabbit anti-IL-12 (1:100), rabbit anti-IRF5 (1:100), rabbit anti-Ym-1 (1:200), and rabbit anti-CD163 (1:100). The sections were subsequently washed and incubated with HRP-conjugated goat anti-rabbit and anti-mouse IgG secondary antibodies, followed by incubation for 5–10 min with 3,3′-diaminobenzidine tetrachloride and visualization of specific staining by light microscopy.

The intensity of collagen III and IV immunostaining in tissue sections was quantified using five representative sections of each slide and determined for five animals in each group, and the area of staining was analyzed as a percentage of the total area. Desmin-positive area was quantified in five random non-overlapping ×100 fields and determined for six animals in each group. The immunostaining signaling was quantified at a fixed threshold using free software NIH ImageJ 1.49 (NIH, Bethesda, MD, United States). For quantification of the numbers of hepatic macrophages in sections, six non-overlapping randomly selected fields of view per slide at ×400 magnifications (F4/80^+^ cells) or ×200 magnifications (CD68^+^, IL-12^+^, CD11c^+^, IRF5^+^, CD163^+^, and Ym-1^+^ cells) were examined and expressed as cells per field of view; and five mice of each group were examined (Miura et al., 2012; Li et al., 2016b, 2017b).

### Immunofluorescence

Details on the immunofluorescence methodology can be found in our previous reports (Li et al., 2017b). Briefly, freshly dissected liver tissues were OCT-embedded and the sections (10 μm in thickness) were cut with a cryotome Cryostat (Leica, 1900, Germany). After blocking in PBS with 3% Bovine serum albumin (BSA), the liver sections were labeled with primary antibody F4/80 (1:100 dilution) overnight at 4°C, and subsequently incubated with antibody Notch1 (1:200 dilution) for 1 h at room temperature (RT) in case of double staining. Alexa Fluor 594 Donkey anti-mouse and Alexa Fluor 488 Donkey anti-rabbit secondary antibodies (Yeasen Biotechnology, Shanghai, China) were incubated at 1:200 in PBS for 1 h at RT. After washing, sections were counterstained with DAPI (4′,6-diamidino-2-phenylindole)-Fluoromount-G^TM^ (SouthernBiotech, United States). Finally, the stained tissues were analyzed by fluorescence microscopy (BX51, Olympus, Japan).

Raw 264.7 cells were fixed and permeabilized in 4% paraformaldehyde, 0.2% TritonX-100 in PBS for 10 min. Nonspecific binding was unmasked with 3% BSA for 1 h at RT, and then the cells were incubated with primary antibodies for IL-12 (dilution 1:200), IRF5 (dilution 1:100), and Notch1 (dilution 1:150) overnight at 4°C. Sections were washed twice with PBS and incubated with fluorescein-labeled secondary antibody at a dilution of 1:500 for 1 h at RT in the dark. Slides were mounted in mounting media with DAPI for 40 min at RT. After washing twice with PBS, the slides were covered with DABCO and images were captured by fluorescence microscopy (IX51, Olympus, Japan).

### Western Blot Analysis

Frozen liver tissue was homogenized in radio immunoprecipitation assay buffer (RIPA buffer) by adding protease inhibitor Cocktail (Roche) and phosphatase inhibitors Cocktail (Sigma, St. Louis, MO, United States), and then centrifuged at 10,000 × *g* at 4°C for 20 min (Li et al., 2016b, 2017a). Protein extraction from Raw 264.7 cells was as previously described (Li et al., 2016b, 2017b). Protein concentration was quantified with the Bicinchoninic Acid Protein Colorimetric Assay kits (BMI, Shanghai, China) with BSA as the standard. Equal amounts of proteins were separated by electrophoresis on 7.5–12% SDS–PAGE gels and transferred onto polyvinylidene difluoride (PVDF) membranes. The membranes were then incubated in blocking buffer [5% nonfat milk powder in tris-buffered saline Tween-20 (TBST)] for 2 h at RT followed by incubation with primary antibody in TBST at 4°C overnight with the specific primary antibodies against Desmin, IRF5, IL-12, and Notch1 (all 1:1000 dilution). The membranes were washed with TBST and then incubated with goat anti-rabbit, anti-mouse, or anti-rat secondary antibodies (1:1500) for 2 h at RT. GAPDH or β-actin (1:5000 dilution) was used as internal control, respectively. After washing off the unbound antibody with TBST, the expression of the antibody-linked protein was determined by an ECL^TM^ Western Blotting Detection Reagents (Amersham Pharmacia Biotech Inc., NJ, United States). The intensity of the western blot bands was performed using NIH ImageJ software. Expression levels were evaluated by quantification of the relative density of each band normalized to that of the corresponding GAPDH or β-actin band density (Li et al., 2016b).

### RNA Isolation and Quantitative RT-PCR

Total RNA was extracted from frozen liver tissue or cultured cells using Trizol reagent (Life Technologies, Grand Island, NY, United States) following manufacturer’s protocol. RNA was reverse-transcribed with random hexamers and avian myeloblastosis virus reverse transcriptase using a commercial kit (Perfect Real Time, SYBR^®^ PrimeScriP^TM^TaKaRa, Japan). Quantitative RT-PCR was performed for assessment of mRNA expression on the ABI Prism 7500 Sequence Detection system (Applied Biosystems, Tokyo, Japan) as previously reported (Li et al., 2017a). Sequences of primers for target genes were purchased from Sangon Biotech Co., Ltd. (Shanghai, China) and listed in **Table 1**. The reactions were run in triplicates using SYBR green gene expression assays. The relative change was normalized to endogenous GAPDH mRNA using the formula 2^-ΔΔC_t_^ (Li et al., 2017a).

**Table 1:** Mouse primer sequences used for quantitative RT-PCR.

Target gene	Forward primers (5′–3′)	Reverse primers (5′–3′)
CTGF	GCGCCTGTTCTAAGACCTGT	TTCATGATCTCGCCATCGGG
Collagen 4α1	AACAACGTCTGCAACTTCGC	CTTCACAAACCGCACACCTG
Collagen 3α1	CCTTCTACACCTGCTCCT	CTTCCTGACTCTCCATCCT
TIMP-1	CCAGAACCGCAGTGAAGAGT	TCTGGTAGTCCTCAGAGCCC
Desmin	CCTACACCTGCGAGATTG	ATCATCACCGTCTTCTTGG
Vimentin	TTCTCTGGCACGTCTTGACC	CTCCTGGAGGTTCTTGGCAG
F4/80	TCTGGGGAGCTTACGATGGA	GAATCCCGCAATGATGGCAC
CD68	GGGGCTCTTGGGAACTACAC	GTACCGTCACAACCTCCCTG
TNFα	GACGTGGAACTGGCAGAAGA	ACTGATGAGAGGGAGGCCAT
IL-1β	GTGCAAGTGTCTGAAGCAGC	CAAAGGTTTGGAAGCAGCCC
IL-6	GGAGTCACAGAAGGAGTGGC	CGCACTAGGTTTGCCGAGTA
MCP-1	AGCCAACTCTCACTGAAGCC	GGACCCATTCCTTCTTGGGG
Notch1	CCTTCGTGCTCCTGTTCTTTGTG	GGGCTCTCTCCGCTTCTTCTTG
NOS2	GAGCAACTACTGCTGGTGGT	CGATGTCATGAGCAAAGGCG
GAPDH	TCTCCTGCGACTTCAACA	TGTAGCCGTATTCATTGTCA
Arg-1	CGTTGTATGATGCACAGCCG	CCCCACCCAGTGATCTTGAC
Ym-1	CTCACTTCCACAGGAGCAGG	AGCTGCTCCATGGTCCTTC

### Statistical Analysis

All data are presented as the mean ±*SD*. Comparisons among multiple groups (three or more) were performed by one-way ANOVA with *post hoc* test (Bonferroni or Dunnett’s correction for multiple tests). For comparison between two groups, the two-tailed unpaired Student’s *t*-test was used for normally distributed data; and the Wilcoxon–Mann–Whitney *U*-tests or Kruskal–Wallis tests were used for non-normally distributed data. Statistical analysis was performed with GraphPad Prism 7.0 (La Jolla, CA, United States). In all comparisons, a *P*-value less than 0.05 was considered as statistically significant.

## Results

### Quercetin Inhibited Liver Inflammation and Fibrosis in CCl_4_-Treated Mice

Remarkably, histological examination revealed that repeated administration of CCl_4_ induced the formation of necrotic areas and inflammation in the liver, with obvious alteration of the sinusoidal and lobular architecture of the liver (**Figure 1B**). However, these morphological changes and inflammation were markedly ameliorated in CCl_4_-induced mice also given oral quercetin (50 mg/kg daily). Control oil-injected mice treated with quercetin did not show any liver injury and inflammation; similar to oil-treated animal administrated with vehicle (**Figure 1B**). Consistent with these results, the necroinflammatory injury score was lower in fibrotic mice treated with quercetin than that in fibrotic mice treated with DMSO (2.7 ± 0.48 vs. 1.9 ± 0.57, *P* < 0.001; **Figure 1D**).

Fibrillar collagen deposition in livers could reflect the severity of fibrosis, which was assessed by examining Sirius red-stained liver sections. Our results revealed that mice-repeated injections of CCl_4_ for 8 weeks induced obviously ECM proteins accumulation, with the formation of bridging fibrosis (**Figure 1C**). While there are only thin layers of collagen surrounded the portal tracts and central veins in the liver from normal control animals. However, fibrotic mice given oral quercetin treatment displayed thinner septa and more preserved hepatic parenchyma than fibrotic animals given vehicle (DMSO) treatment (**Figure 1C**). Furthermore, collagen deposition in the liver of CCl_4_-treated mice was confirmed by computerized image analysis of the fibrotic area, whereas fibrotic mice treated with quercetin markedly attenuated the progression of CCl_4_-induced fibrosis when compared with vehicle-treated fibrotic mice (4.93 vs. 11.22%, *P* < 0.001; **Figure 1E**). Similarly, we observed that the mean fibrosis score was significantly lower in fibrotic mice also given quercetin treatment than that in fibrotic mice given vehicle treatment (2.3 ± 0.8 vs. 3.5 ± 0.7, *P* < 0.001; **Figure 1F**).

**FIGURE 2:**
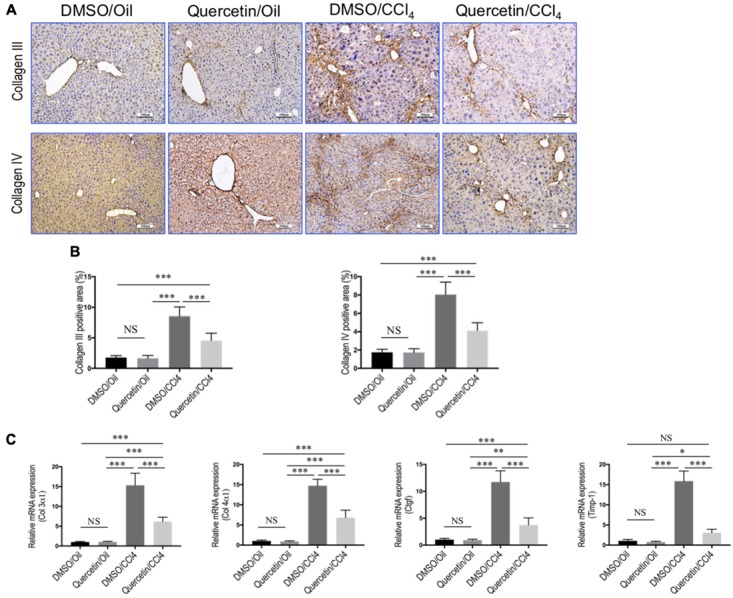
Quercetin inhibited liver fibrotic markers expression in CCl_4_-induced mouse fibrotic liver model. **(A)** Representative microscopy images of Collagen III and Collagen IV immunohistochemistry in the liver (original magnification, ×100). Scale bar = 100 μm. **(B)** Quantitative analysis of Collagen III- and Collagen IV-positive area by ImageJ software (NIH). *n* = 5/group. **(C)** Expression of fibrotic markers (*Col3 α1*, *Col4α1*, *Ctgf*, and *Timp-1*) was examined by quantitative RT-PCR in whole liver samples from each group (*n* = 6/group). Results were normalized to GAPDH mRNA and expressed as fold change compared to DMSO/oil mice. *^∗^P* < 0.05, ^∗∗^*P* < 0.01, ^∗∗∗^*P* < 0.001; “NS” indicates not significant.

Additionally, immunohistochemical evaluation revealed that the deposition of intrahepatic collagen III and IV was increased in fibrotic mice induced by CCl_4_ for 8 weeks, whereas co-treatment with quercetin attenuated these collagen accumulations in the liver when compared with DMSO-treated control (**Figure 2A**). These results were further confirmed by quantification of collagen III or collagen IV immunopositive areas; indicating that fibrotic mice treated with quercetin significantly reduced the deposition of collagen in the liver when compared with vehicle-treated control animal (**Figure 2B**). Moreover, we also assessed the expression levels of the markers of profibrogenic genes, such as *Col3α1*, *Col4α1*, connective tissue growth factor (*Ctgf*), and tissue inhibitor of metalloproteinase-1 (*Timp-1*). We found that the levels of those profibrogenic genes were observably enhanced in CCl_4_-induced mice when compared with oil-treated normal control; however, quercetin treatment obviously inhibited the profibrogenic effects of CCl_4_ injection and decreased the abundance of these genes expression as compared to vehicle-treated animals (**Figure 2C**).

Taken together, these results indicated that quercetin strikingly attenuated liver inflammation and fibrogenesis in CCl_4_-induced liver fibrosis mouse model.

### Quercetin Inhibited Activation of HSCs in CCl_4_-Treated Mice

**FIGURE 3:**
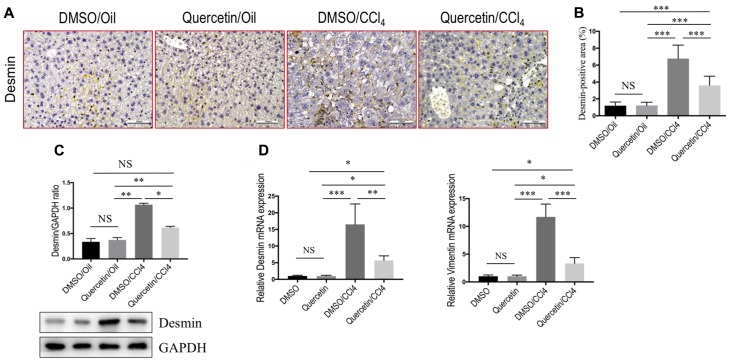
Quercetin inhibited hepatic stellate cells (HSCs) activation in CCl_4_-treated mice. **(A)** Representative microscopy images of desmin staining (magnification: ×200) in the liver. Scale bar = 100 μm. **(B)** Quantification of desmin-positive area by ImageJ software (NIH). Results mean of six fields and *n* = 5/group. **(C)** Western blotting analysis of desmin expression in lysed liver tissues, with results normalized relative to the expression of GAPDH (*n* = 3). **(D)** Expression of desmin and vimentin mRNA was determined in the liver by quantitative RT-PCR (*n* = 6). Results were normalized relative to GAPDH expression and expressed as mean ±*SD* fold change over normal control mice. ^∗^*P* < 0.05; ^∗∗^*P* < 0.01; ^∗∗∗^*P* < 0.001; “NS” indicates not significant.

In order to investigate whether quercetin affects the activation of HSCs in the liver, we examined the expression of HSC-specific marker with immunohistochemical (IHC) staining. In our previous study, we demonstrated that quercetin inhibited α-SMA expression at gene and protein level both *in vivo* and *in vitro* (Li et al., 2016b). To provide additional support evidence, we here examined other HSCs activated markers such as desmin and vimentin (Bansal et al., 2015; Wilhelm et al., 2016). Indeed, as revealed by immunostaining, there were markedly strong desmin signals in the fibrotic septa in the CCl_4_-induced livers, while only faint staining for desmin in livers from normal mice; however, there was relatively weak intensity of desmin staining in livers from fibrotic mice receiving quercetin treatment when compared with those fibrotic mice receiving DMSO treatment (**Figure 3A**). Furthermore, computer-assisted semi-quantitative analysis showed that the number of desmin-positive cells was markedly decreased in livers from quercetin-treated fibrotic mice than those from vehicle-treated control mice (3.52 ± 0.16 vs. 7.83 ± 0.23%, *P* < 0.001; **Figure 3B**). These results were also confirmed by western blot analysis and quantitative RT-PCR experiments, indicating that there was lower expression in the levels of desmin gene and protein after chronic CCl_4_ mice receiving quercetin compared with those mice receiving vehicle (**Figures 3C,D**). In addition, there was a corresponding reduction in mRNA expression levels of vimentin (**Figure 3D**).

Collectively, these findings indicated that quercetin treatment efficiently reduced HSC-derived myofibroblasts activation in mice induced by CCl_4_.

### Quercetin Inhibited Massive Macrophages Recruitment into the Fibrotic Livers of CCl_4_-Induced Mice

**FIGURE 4:**
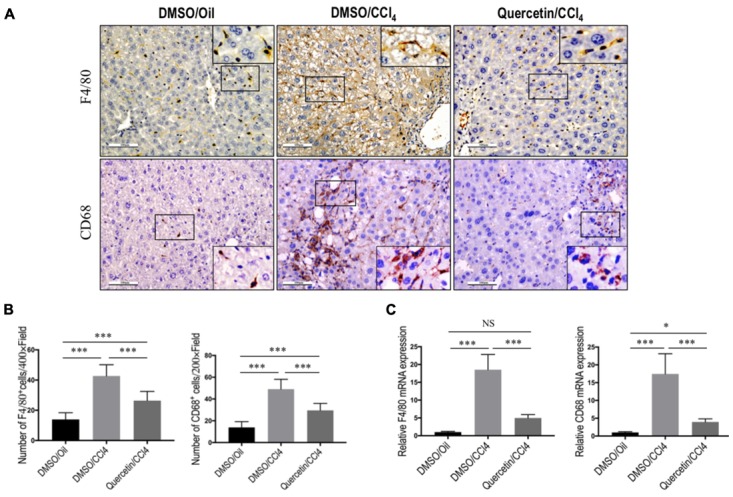
Quercetin inhibited massive macrophage recruitment into the fibrotic livers of CCl_4_-induced mice. **(A)** Immunohistochemical detection of F4/80- and CD68-positive cells in liver sections from each group (original magnification: ×200). Insert (magnification: ×400) shows typical morphology of positive macrophages. Scale bar = 100 μm. **(B)** Quantification of F4/80- and CD68-positive cells in liver sections. Results mean of six fields and *n* = 5/group. **(C)** Gene expression of macrophage marker F4/80 and CD68 was determined in livers by quantitative RT-PCR, and the results are shown as fold change compared with sham-treated control and GAPDH served as loading control (*n* = 6). ^∗^*P* < 0.05; ^∗∗∗^*P* < 0.001; “NS” indicates not significant.

To assess whether the effects of quercetin on liver fibrogenesis were related to the infiltration of macrophages in livers, liver sections were stained with antibodies against macrophage markers, F4/80 and CD68. IHC staining results revealed a higher number of F4/80^+^ or CD68^+^ cells in livers after chronic CCl_4_ damage compared to the normal control livers (**Figure 4A**). Remarkably, these positive macrophages were predominantly observed in the scars of fibrotic livers. However, the number of macrophages infiltration in livers was markedly reduced in fibrotic mice receiving quercetin treatment when compared with those mice given DMSO treatment (**Figures 4A,B**). This observation was further verified by quantification of the F4/80^+^ or CD68^+^ staining cells, indicating that repeated CCl_4_ injection for 8 weeks significantly promoted macrophages recruitment into the livers, and that the increased number of macrophages was significantly lower in CCl_4_-induced mice with quercetin treatment when compared with vehicle-treated fibrotic control (**Figures 4A,B**). Consistent with these IHC findings, mRNA levels of F4/80 and CD68 in total liver also demonstrated that quercetin treatment to fibrotic mice blocked the up-regulated F4/80 and CD68 expression (**Figure 4C**). Taken together, these findings suggested that quercetin treatment significantly reduced massive hepatic macrophage recruitment to the injured liver.

### Quercetin Inhibited M1 Macrophage Polarization and Expression of Inflammatory Properties in Fibrotic Livers

**FIGURE 5:**
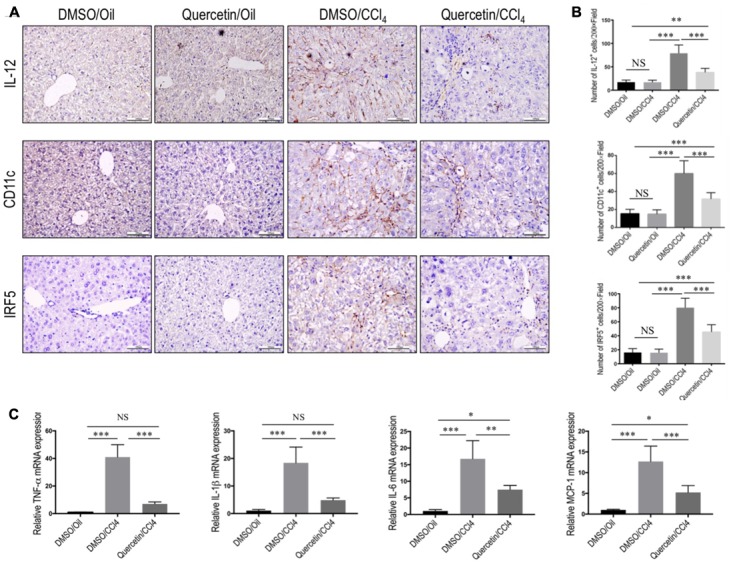
Effect of quercetin on M1 macrophage polarization and expression of inflammatory properties in fibrotic livers. **(A)** Representative immunostaining of IL-12, CD11c, and IRF5 in liver sections (original magnification: ×200). Scale bar = 100 μm. **(B)** Quantification of IL-12, CD11c, and IRF5 immunostaining in the liver from each group. **(C)** Hepatic M1 polarization genes of TNF-α, IL-1β, IL-6, and MCP-1 were determined by quantitative RT-PCR, and results are shown as fold change compared with sham-treated control and GAPDH served as loading control (*n* = 5). ^∗^*P* < 0.05; ^∗∗^*P* < 0.01; ^∗∗∗^*P* < 0.001; “NS” indicates not significant.

To determine whether quercetin restricts hepatic injury, inflammation, and fibrosis through switching macrophages phenotype and influencing its function, we assessed the status of M1 macrophages and expression of proinflammatory cytokines associated with M1 markers in the fibrotic liver [such as TNF-α, IL-1β, IL-6, and monocyte chemotactic protein-1 (MCP-1)]. Of note, IL-12, an important cytokine produced by classically activated macrophages, could be served as an IHC marker to detect the M1-dominant subset in livers (Beljaars et al., 2014); and other M1 macrophage markers were strongly confirmed by previous studies including CD11c and IRF5 (Beljaars et al., 2014; Alzaid et al., 2016). We found that chronic CCl_4_ injection increased M1 macrophages, as determined by IHC staining with antibodies against CD11c, IL-12, and IRF5 (**Figure 5A**) and by quantification of CD11c^+^ cells, IL-12^+^ cells, and IRF-5^+^ cells in liver sections (**Figure 5B**), while these positive cells were restricted to liver sinusoids and fibrotic septa in CCl_4_-induced fibrotic mice, and barely detectable in healthy livers; however, quercetin-treated CCl_4_ mice suppressed M1 macrophages polarization as confirmed by M1-associated markers (**Figures 5A,B**). Notably, these macrophages were found solely in the fibrotic collagen bands. We also confirmed these results via quantitative RT-PCR and found that repeated CCl_4_ injection has been associated with enhanced proinflammatory cytokine markers in the liver, including TNF-α, MCP-1, IL-6, and IL-1β mRNA, as compared with the normal control. However, compared with fibrotic mice receiving DMSO treatment, fibrotic mice receiving quercetin decreased the levels of TNF-α, IL-1β, IL-6, and MCP-1 mRNA expression by 4.85-, 2.78-, 1.24-, and 1.43-fold, respectively (**Figure 5C**).

Taken together, these results show that quercetin inhibited M1 polarization of macrophage and reduced the expression of inflammatory properties in fibrotic livers following repeated injection CCl_4_.

### Quercetin Attenuated M2 Macrophages Polarization and Expression of Immunosuppressive Genes in Fibrotic Livers

**FIGURE 6:**
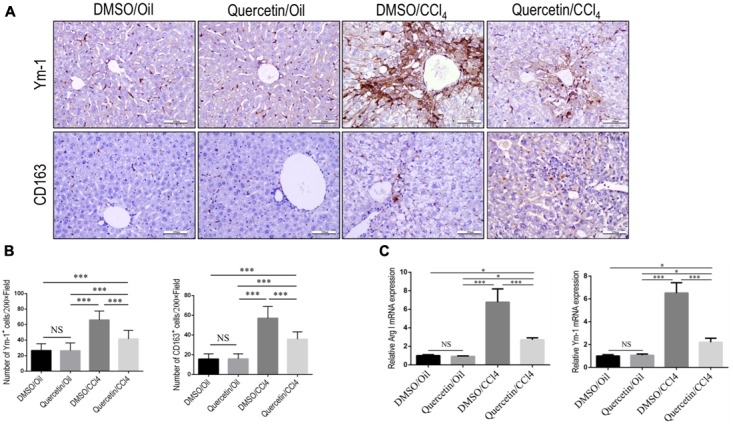
Effect of quercetin on M2 macrophages polarization and expression of immunosuppressive genes in fibrotic livers. **(A)** Representative immunostaining of Ym-1 and CD163 in liver sections (original magnification: ×200). Scale bar = 100 μm. **(B)** Quantification of Ym-1 and CD163 immunostaining in the liver from each group. **(C)** M2-polarized gene expression of Arginase I (Arg I) and Ym-1 was determined by quantitative RT-PCR, and the results are shown as fold change compared with sham-treated control and GAPDH served as loading control (*n* = 5). ^∗^*P* < 0.05; ^∗∗∗^*P* < 0.001; “NS” indicates not significant.

In addition, we also evaluated the effect of quercetin on M2-polarized macrophages and activation in the development of liver fibrosis *in vivo*. We used chitinase-3-like 3 (Chi3l3; also known as Ym-1; mouse only) and CD163 (Beljaars et al., 2014; Alzaid et al., 2016) as molecular M2 macrophage markers; and our results demonstrated that the expression of M2 markers in livers was clearly higher after chronic CCl_4_ damage as demonstrated by immunostaining with antibodies against Ym-1 and CD163 (**Figure 6A**); however, mice given quercetin decreased those M2 macrophages staining signaling when compared with the vehicle-treated mice (**Figure 6A**). These results were further confirmed by quantification of the Ym-1^+^ and CD163^+^ staining cells (**Figure 6B**), indicating quercetin-treatment obviously reduced the M2 macrophages recruitment into the liver following 8-week CCl_4_ administration. Moreover, the M2 skewing was further confirmed with quantitative RT-PCR for selective M2 typical markers such as Arginase I (Arg I) and Ym-1; and we observed the levels of M2 marker genes (Arg I and Ym-1) in fibrotic livers decreased by quercetin-treated fibrotic mice when compared with DMSO-treated animals (**Figure 6C**). Together, these results show that quercetin inhibited M2-dominant macrophage polarization in fibrotic livers and limited immunosuppressive genes following CCl_4_ administration in mice.

### Quercetin Treatment Suppressed M1-Polarized Macrophages *in Vitro*

**FIGURE 7:**
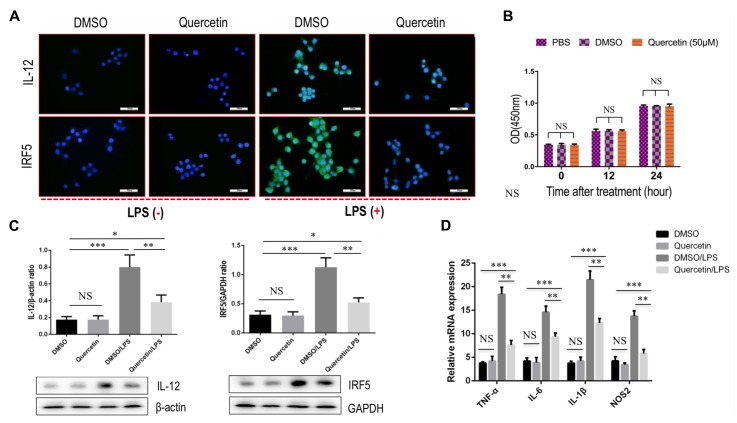
Quercetin treatment suppressed M1 polarization of macrophages *in vitro.* **(A)** Representative fluorescence microscopic images of RAW macrophages with anti-IL12 and anti-IRF5 whole-mount staining. Undifferentiated RAW macrophages conditioned medium, and using LPS (100 ng/mL) to induce M1 differentiation. Quercetin- (50 μM) or DMSO-treated M1-differentiated macrophages conditioned medium. Bars represent mean ±*SD* of at least three independent experiments. Scale bar = 200 μm. **(B)** Effect of quercetin (50 μM) on the cell viability of macrophages. Cell viability was then determined by the CCK-8 assay as described in the “Materials and Methods” section. **(C)** Western blotting analysis of M1-markers IL12 and IRF5 protein expression in macrophages RAW 264.7 cells, with results normalized relative to the expression of β-actin or GAPDH (*n* = 3). **(D)** Quantification gene expression analysis of M1-specific markers TNF-α, IL-1β, IL-6, and NOS2. The mRNA levels were normalized to GAPDH mRNA levels and presented as fold stimulation (mean ± *SD*) versus vehicle-treated control. ^∗^*P* < 0.05; ^∗∗^*P* < 0.01; ^∗∗∗^*P* < 0.001; “NS” indicates not significant.

To further interrogate whether quercetin treatment may prevent M1-polarized macrophages in fibrotic liver, we used the RAW 264.7 cell line as the model of M1 macrophages *in vitro*, as the cells can be reliably polarized to M1 macrophages *in vitro* by stimulation with LPS (Wan et al., 2014; Tosello-Trampont et al., 2016; Labonte et al., 2017). Indeed, our experiment demonstrated that incubation with quercetin to the cells markedly suppressed M1-macrophages polarization as shown in immunostaining with anti-IL12 and anti-IRF5 (**Figure 7A**). Notably, we observed that quercetin, at this dosage, did not affect the cell viability of macrophages *in vitro* (**Figure 7B**). We then examined the expression of M1 macrophages markers such as IL12 and IRF5 by western blotting; our results demonstrated that quercetin treatment significantly decreased the levels of those markers expression on macrophages when compared with vehicle-treated cells (**Figure 7C**). Furthermore, we also revealed that quercetin led to the substantially reduced M1-polarized macrophages as depicted in M1-related markers such as *TNF*-α, *IL-1*β, *IL-6*, and nitric oxide synthase 2 (*NOS2*) (**Figure 7D**). Taken together, these data suggested that quercetin treatment may regulate the M1-polarized macrophages upon injury *in vitro*.

### Quercetin Inhibited Hepatic Macrophages Activation and Suppressed M1-Polarization through Regulating the Expression Notch1 on Macrophages

**FIGURE 8:**
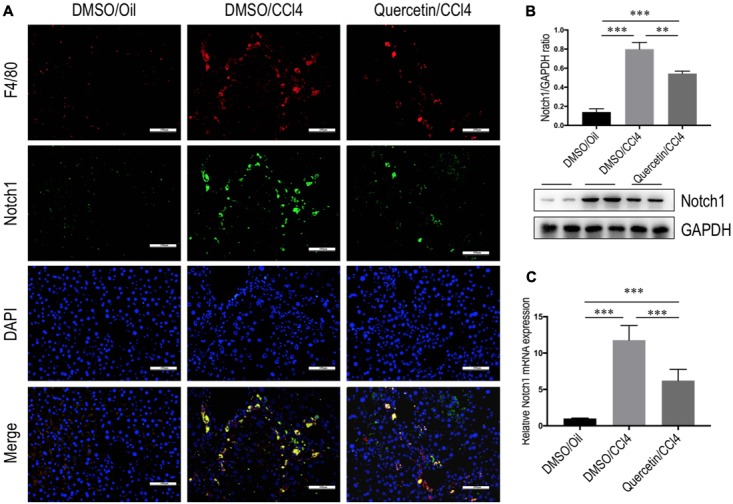
Quercetin inhibited hepatic Notch1 expression in CCl_4_-treated mice. **(A)** Immunofluorescent double staining of Notch1 in liver sections from each group. Livers were double stained for Notch1 (green) and F4/80 (Red) macrophages; DAPI as blue nuclear counterstain. Scale bar = 100 μm. **(B)** Western blotting analysis of Notch1 expression in lysed liver tissues, with results normalized relative to the expression of GAPDH (*n* = 3). **(C)** Hepatic Notch1 mRNA expression was measured by quantitative RT-PCR. Results are shown as fold change compared with oil-treated control and GAPDH served as loading control (*n* = 5). ^∗∗^*P* < 0.01; ^∗∗∗^*P* < 0.001.

Recent data have suggested that Notch1 signaling was widely known as a key transcription factor related to M1 macrophage activation (Lawrence and Natoli, 2011; Bansal et al., 2015; Xu et al., 2015a). To verify the involvement of quercetin in regulating M1 macrophages in liver fibrogenesis through Notch1 signaling pathway, we first examined the Notch1 expression and subcellular location in the liver. Double-staining of liver sections from fibrotic mice showed that Notch1 was localized predominantly in resident macrophages (F4/80), the staining signal of Notch1 in fibrotic liver is stronger than oil-treated normal liver (**Figure 8A**). Then, we assessed the expression of Notch1 in livers from each group by quantitative RT-PCR and western blots. Our data revealed that the levels of Notch1 expression in fibrotic livers were marked increase when compared with those in the normal control livers; however, quercetin-treated fibrotic mice decreased the levels of Notch1 gene and protein expression when compared with vehicle-treated fibrotic animals (**Figures 8B,C**).

**FIGURE 9:**
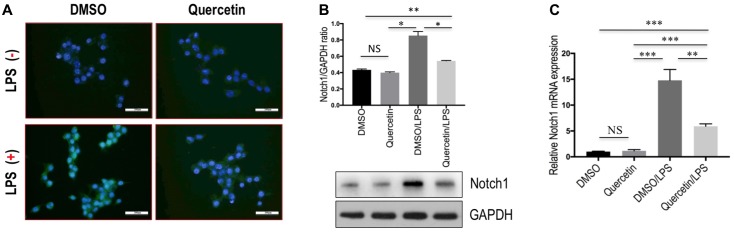
Quercetin inhibited M1-macrophages polarization through regulating the expression Notch1 on macrophages *in vitro*. **(A)** Undifferentiated RAW 264.7 macrophages conditioned medium or LPS-stimulated M1-differentiated macrophages, quercetin (50 μM)-treated RAW macrophages, DMSO as vehicle-treated control. Scale bar = 200 μm. **(B)** Western blotting analysis for Notch1 in RAW 264.7 cells. **(C)** The levels of Notch1 mRNA expression in RAW 264.7 cells were measured by quantitative RT-PCR. The mRNA levels were normalized to GAPDH mRNA levels and presented as fold stimulation (mean ±*SD*) versus DMSO. ^∗^*P* < 0.05; ^∗∗^*P* < 0.01; ^∗∗∗^*P* < 0.001; “NS” indicates not significant.

Finally, we determined whether quercetin inhibits M1 polarization macrophages through regulating Notch1 expression on macrophages *in vitro*. RAW 264.7 macrophages were incubated with quercetin prior to induction of M1-polarized macrophages. The results showed that the expression of Notch1 was increased in RAW 264.7 cells by treatment with LPS, whereas quercetin (50 μM) in combination to treatment cells significantly abrogated the increase of Notch1 gene and protein expression, as measured by immunofluorescence (**Figure 9A**), western blots (**Figure 9B**), and quantitative RT-PCR (**Figure 9C**). Moreover, these alterations in expression of Notch1 are paralleled by the reduced genetic expression of the M1-specific markers in macrophages, such as IL-1β, IL-6, and NOS2 (**Figure 7D**). Collectively, these results indicated that quercetin inhibited M1-porlizated macrophages via targeting Notch1.

## Discussion

In this study, we have provided both *in vivo* validation and mechanistic insights regarding the protective effects of the flavonoid quercetin in CCl_4_-induced liver injury and fibrosis in mice. Importantly, our current finding provides a novel insight for understanding the antifibrotic activity of quercetin, which owing to its inhibition of hepatic macrophages activation and infiltration, and modulation of M1-polarized macrophages via the Notch1 pathway. In addition, we also have strongly reinforced the notion that hepatic macrophages play a critical role in the development of liver fibrosis, and strategies restraining M1 macrophage polarization phenotype may protect against exacerbated inflammation and thus restrict liver fibrosis (Sica et al., 2014; Tacke and Zimmermann, 2014; Tacke, 2017).

Quercetin, a polyphenol (diferuloylmethane), has manifested a diverse range of pharmacological activities including anti-inflammatory, antioxidant, antibacterial, and antitumor properties (Russo et al., 2012; Wu et al., 2017). In the present experiment, using the well-established liver fibrosis model by injection CCl_4_ in mice, we provided more evidence that quercetin ameliorated liver inflammation and fibrosis (**Figure 1**). In liver fibrogenesis, excess ECMs, including collagen, are mainly produced by activated HSCs (Friedman, 2008; Tsuchida and Friedman, 2017). Our results also demonstrated that quercetin inhibited the activation of HSCs by CCl_4_ intoxication (**Figure 3**). Moreover, when we examined collagen synthesis by measuring the levels of expression of mRNA encoding Collagen α3(I) and Collagen α4(I), and the activated HSC markers, we found that the expression levels of these fibrogenic markers were markedly lower in quercetin- than in vehicle-treated mice after 8 weeks of CCl_4_ injection (**Figure 2C**). These data are consistent with the observation that quercetin treatment inhibited HSC activation *in vitro* as shown for the expression of genetic markers such as Collagen α1(I), TGF-β1, and α-SMA (Li et al., 2016b). Furthermore, the data demonstrated that the mechanism of inhibition of liver fibrosis by quercetin was surmised to be direct downregulation of the HMGB1–TLR2/4-NFκB signaling pathway (Li et al., 2016b). Recently, an interesting study demonstrated that quercetin restricted liver fibrosis by inhibiting HSC activation and reducing autophagy through regulating crosstalk between the TGF-β1/Smads and PI3K/Akt pathways (Wu et al., 2017). Here, we provided evidence that quercetin inhibited liver fibrosis through regulating macrophage polarization and function via Notch1 pathway.

Emerging data have recently demonstrated that macrophages play a complex role in liver fibrogenesis, involved in progression and resolution of hepatic fibrosis (Sica et al., 2014; Tacke and Zimmermann, 2014; Tacke, 2017). Inflammatory cytokines released from those cells perpetuate inflammation as well as activating HSCs (Pradere et al., 2013; Pellicoro et al., 2014; Tacke, 2017). In this study, we demonstrated that quercetin reduced hepatic macrophage number and ameliorated liver fibrosis following CCl_4_ treatment (**Figure 4**). Notably, our data further suggested that quercetin suppressed M1-polarized macrophages that have inflammatory properties and mediate excessive liver inflammation and fibrosis (**Figure 5**). Consistent with the inhibition in M1 macrophages activation and shift, the inflammatory cytokines were decreased in quercetin-treated fibrotic livers and in quercetin-treated macrophages when compared with the respective controls (**Figure 5C**). In order to investigate the effect of quercetin on M1-polarized macrophages, we used Raw 264.7 cell line as *in vitro* model to study. We found that quercetin indeed blocked LPS-mediated M1 macrophages activation as measured by immunofluorescence, western blots, and quantitative RT-PCR (**Figure 7**). Therefore, our data indicated that quercetin could serve as a regulator of macrophage recruitment and polarization in injury liver.

We also assessed the effect of quercetin on M2-polarized macrophages in fibrotic livers in mice induced by CCl_4_ for 8 weeks. Our results showed that the number of Ym-1^+^ and CD163^+^ macrophages in fibrotic livers was obviously increased with displayed higher hepatic expression of M2-macrophage genes (Arg I and Ym-1), as compared to the normal control mice. However, treatment of fibrotic mice with quercetin inhibited M2 macrophages polarization and decreased expression of classic M2 genes in fibrotic livers (**Figure 6**). On the contrary, previous *in vitro* study has demonstrated that quercetin could induce M2 polarized macrophages (Dong et al., 2014). It is worth to note that inactivation of the M2 macrophages contributed to diet-induced NASH *in vivo* studies and data have recently demonstrated that M2-polarized macrophages promote resolution of inflammation and tissue repair (Beljaars et al., 2014; Tacke, 2017). Consist with our results, previous studies have also demonstrated that Ym-1^+^, CD206^+^, or CD163^+^ macrophages were increased and had the potential effect on liver inflammatory changes in different liver inflammation and fibrosis animal models (Beljaars et al., 2014; Ohtsuki et al., 2015; Svendsen et al., 2017). Additionally, a recent report suggests that dietary quercetin ameliorated high-fat diet-induced obesity and insulin resistance in mice by regulating the balance of M1/M2 polarization in liver macrophages and reducing the levels of proinflammatory cytokines (Dong et al., 2014). Those different results indicated there is remarkable heterogeneity of liver macrophages with diverse functions, and that function varies according to the phage of injury and depending on the hepatic microenvironment, and is also influenced by the nature of the underlying liver injury (Tacke and Zimmermann, 2014; Tacke, 2017). In addition, there certainly existed functionally distinct macrophage subtypes, which are not simply a subpopulation of macrophages (CD163^+^ or Ym-1^+^) in this study that could be explained the role of M2 macrophages in the development of pathological fibrosis. Thus, understanding of macrophage polarization and function is a keystone of deciphering liver fibrogenesis. Future studies are needed to further confirm whether quercetin mediated the M2 macrophages polarization both *in vivo* and *in vitro*.

Recent investigations have focused on elucidating the molecular mechanisms that suppress inflammation and prevent the development of fibrosis (Wynn and Vannella, 2016). It has revealed that macrophage differentiation and activation is subjected to tight control by several mechanisms, including signaling molecules, transcription factors, epigenetic mechanisms, and posttranscriptional regulators (Lawrence and Natoli, 2011; Wynn et al., 2013; Xu et al., 2015b). Of note, emerging evidence has suggested that Notch pathway plays an important role in macrophage-mediated inflammation (Palaga et al., 2008; Xu et al., 2015b; Kimball et al., 2017). A recent study demonstrated that Notch1-mediated signaling regulation of M1 macrophage activation contributed to the inflammatory pathologies in alcoholic liver disease and obesity-induced liver disease (Xu et al., 2015a,b). It has also been reported that suppressor of cytokine signaling 3 (SOCS3) may play an important role in Notch signaling-mediated M1 macrophage polarization (Eun and Jeong, 2016). In this study, we demonstrated that Notch1 expression on macrophages was increased during liver injury in mice; however, quercetin treatment abrogated the increased level of Notch1 expression (**Figure 8**). Additionally, in the presence of LPS-induced macrophage activation *in vitro*, in line with M1 polarization, the expression of Notch1 on macrophages was increased. However, quercetin treatment inhibited M1 polarization and the Notch1 expression on macrophages (**Figure 9**). Of note, we have recently demonstrated that quercetin could inhibit HMGB1–TLR2/TLR4-NFkB signaling pathway in the fibrotic liver (Li et al., 2016b). Given that TLR2/TLR4 signaling was also involved in proinflammatory macrophages activation (Palaga et al., 2008; Kimball et al., 2017), we deduced that this inhibitory effect of quercetin might partially mediate in suppressing M1 polarization of macrophages. Collectively, our data highlight a key role for the Notch1 pathway in regulating M1 macrophage polarization in liver injury and fibrosis, indicating that blockade of Notch1 signaling may represent a promising therapeutic target for chronic liver inflammation and fibrosis.

## Conclusion

Our data lead the evidence for supporting the concept that the hepatic macrophages play a key role in the development of liver fibrosis; and quercetin treatment could be a potential agent for chronic liver inflammation and fibrosis, at least in part, by manipulating macrophage phenotype and activation. This quercetin’s antifibrotic activity should be studied in treating human chronic liver diseases in future.

## Ethics Statement

The present study was approved by the Animal Care Committee of Fudan University (Shanghai, China).

## Author Contributions

XL and CT conceived the study and wrote the manuscript. XL, SZ, and CT contributed to the work designing, performing, analyzing, and interpreting data from all the experiments. QY, QJ, SZ, and LL participated in the design, acquisition, analysis, and interpretation of the data. CT and XL carried out the animal model and all the *in vivo* animal experiments. CT, SZ, and XL interpreted the data and finalized the article. All authors have critically revised and approved the final manuscript and agreed to be accountable for all aspects of the work.

## Conflict of Interest Statement

The authors declare that the research was conducted in the absence of any commercial or financial relationships that could be construed as a potential conflict of interest.
